# Metabolic engineering of *Saccharomyces cerevisiae* for production of β-carotene from hydrophobic substrates

**DOI:** 10.1093/femsyr/foaa068

**Published:** 2020-12-17

**Authors:** Zahra Fathi, Larissa Ribeiro Ramos Tramontin, Gholamhossein Ebrahimipour, Irina Borodina, Farshad Darvishi

**Affiliations:** Department of Microbiology and Microbial Biotechnology, Faculty of Life Sciences and Biotechnology, Shahid Beheshti University, Tehran, Iran; The Novo Nordisk Foundation Center for Biosustainability, Technical University of Denmark, Denmark; Department of Microbiology and Microbial Biotechnology, Faculty of Life Sciences and Biotechnology, Shahid Beheshti University, Tehran, Iran; The Novo Nordisk Foundation Center for Biosustainability, Technical University of Denmark, Denmark; Department of Microbiology, Faculty of Biological Sciences, Alzahra University, Tehran, Iran; Microbial Biotechnology and Bioprocess Engineering (MBBE) Group, Department of Microbiology, Faculty of Science, University of Maragheh, Maragheh, Iran

**Keywords:** carotenoids, lipases, *Yarrowia lipolytica*, *Saccharomyces cerevisiae*, oil, cell factories

## Abstract

β-Carotene is a yellow–orange–red pigment used in food, cosmetics and pharmacy. There is no commercial yeast-based process for β-carotene manufacturing. In this work, we engineered the baker's yeast *Saccharomyces cerevisiae* by expression of lipases and carotenogenic genes to enable the production of β-carotene on hydrophobic substrates. First, the extracellular lipase (*LIP*2) and two cell-bound lipases (*LIP*7 and *LIP8*) from oleaginous yeast *Yarrowia lipolytica* were expressed either individually or in combination in *S. cerevisiae*. The engineered strains could grow on olive oil and triolein as the sole carbon source. The strain expressing all three lipases had ∼40% lipid content per dry weight. Next, we integrated the genes encoding β-carotene biosynthetic pathway, *crtI*, *crtYB* and *crtE* from *Xanthophyllomyces dendrorhous*. The resulting engineered strain bearing the lipases and carotenogenic genes reached a titer of 477.9 mg/L β-carotene in yeast peptone dextrose (YPD) medium supplemented with 1% (v/v) olive oil, which was 12-fold higher than an analogous strain without lipases. The highest β-carotene content of 46.5 mg/g DCW was obtained in yeast nitrogen base (YNB) medium supplemented with 1% (v/v) olive oil. The study demonstrates the potential of applying lipases and hydrophobic substrate supplementation for the production of carotenoids in *S. cerevisiae*.

## Teaser

Metabolic engineering of *Saccharomyces cerevisiae* using synthetic biology tools for production of β-carotene from hydrophobic substrates.

## INTRODUCTION

Carotenoids are tetraterpenoid pigments, naturally produced by some plants, algae, fungi and bacteria. There are over a thousand known carotenoids. They fall into two classes, oxygen-containing xanthophylls and carotenes that do not contain oxygen. Commercially relevant carotenoids include β-carotene, lycopene, astaxanthin, zeaxanthin, cantaxanthin, lutein and some others. The carotenoid market was estimated at $1.5 billion in 2017 and is projected to grow at a compound annual growth rate of 5.7% (https://www.bccresearch.com/market-research/food-and-beverage/the-global-market-for-carotenoids.html). Biologically produced β-carotene represents a mix of stereoisomers, while chemically synthesized β-carotene is the trans-isomer only. Most of β-carotene is currently produced by chemical synthesis. The other methods include extraction from palm oil or carrots and fermentation of natural producers, such as *Dunaliella* spp. algae or filamentous fungus *Blakeslea trispora*. Recently, novel recombinant β-carotene producing yeast strains have been developed. Some of the highest titers (4–6.5 g/L) were reported for oleaginous yeast *Yarrowia lipolytica* (Gao *et al*. 2017; Larroude *et al*. 2017), with β-carotene content of 90 mg/g DCW. Increasing lipid accumulation in *Y. lipolytica* was an effective strategy to improve the production of β-carotene, likely due to improved storage of β-carotene in lipid bodies (Gao *et al*. 2017; Larroude *et al*. 2017). In contrast to *Y. lipolytica*, *S. cerevisiae* does not naturally accumulate high amounts of lipids and it also has a low formation of cytosolic acetyl-CoA, which is a precursor for both lipids and β-carotene. However, *S. cerevisiae* is a widely used industrial host for advanced biofuels, recombinant proteins, organic acids and fine chemicals (Li and Borodina 2015). A commercial process for the production of lycopene in *S. cerevisiae* has recently been established and there is interest to use this yeast for the production of other carotenoid pigments as well (Ma *et al*. 2019). As the host for carotenoid production, *S. cerevisiae* has several advantages. It is the most studied yeast species, with sequenced and well-annotated genome and excellent genome editing tools (Stovicek, Holkenbrink and Borodina 2017). It is well amenable for large-scale cultivations and has a long history of safe use in food and feed applications. It has been previously engineered for a high-level production of several terpenoid compounds, such as farnesene, bisabolene and artemisic acid (Ro *et al*. 2006; Peralta-Yahya *et al*. 2011; Tippmann *et al*. 2016). Multiple studies have reported engineering of *S. cerevisiae* strains for the production of β-carotene (Verwaal *et al*. 2007; Wang *et al*. 2019; Sun *et al*. 2020). Interestingly, the highest titer of ∼770 mg/L was obtained in fed-batch fermentation with xylose as carbon source and the strain SR8B did not feature any modifications of the mevalonate pathway (Sun *et al*. 2020). The content of β‐carotene was 11.4 mg/g DCW. Sun *et al*. (2016) observed that in a *S. cerevisiae* strain engineered for β-carotene production, the cellular content of ergosterol and unsaturated fatty acids decreased. Supplementation of the cultivation medium with 60 mg/L oleic acid and palmitoleic acid restored the intracellular content of unsaturated fatty acids and also resulted in increased β-carotene content, by 83.7% and 130.2%, respectively (Sun *et al*. 2016).

We hypothesized that supplementing *S. cerevisiae* with fatty substrates, e.g. lipids, may enhance β-carotene accumulation through several mechanisms. Firstly, some of the lipids would accumulate in the cells, providing the lipophilic environment for carotenoids accumulation. Secondly, part of the lipids would be metabolized in the cells via β-oxidation pathway, generating acetyl-CoA, the precursor for β-carotene. The cost of plant oils and fats is two-three orders of magnitude lower than the cost of carotenoids, hence it would not be economically prohibitive to add these feedstocks to the fermentation medium. Furthermore, some oil-containing wastes, such as olive mill waste, are negative-cost substrates (Probst *et al*. 2016). While free fatty acids are toxic to yeast cells at high concentrations (Eisenberg and Büttner 2014), oils or fats do not exhibit toxicity. However, wild-type isolates of *S. cerevisiae* are unable to produce any extracellular lipase and therefore are not capable of utilizing lipids efficiently (Ciafardini, Zullo and Iride 2006).

Therefore, heterologous lipases are required. We decided to use oleaginous yeast *Y. lipolytica* as the source of lipases (Darvishi *et al*. 2017). This yeast produces several extracellular, cell-bound and intracellular lipases. There are 16 lipase encoding genes in the genome sequence of *Y. lipolytica* strain E150: *LIP2* (YALI0A20350g), *LIP4* (YALI0E08492g), *LIP5* (YALI0E02640g), *LIP7* (YALI0D19184g), *LIP8* (YALI0B09361g), *LIP9* (YALI0E34507g), *LIP10* (YALI0F11429g), *LIP11* (YALI0D09064g), *LIP12* (YALI0D15906g), *LIP13* (YALI0E00286g), *LIP14* (YALI0B11858g), *LIP15* (YALI0E11561g), *LIP16* (YALI0D18480g), *LIP17* (YALI0F32131g), *LIP18* (YALI0B20350g) and *LIP19* (YALI0A10439g) (http://www.genolevures.org/fam/GL3R0084; Casaregola *et al*. 2000; Fickers, Marty and Nicaud 2011). Extracellular lipase activities have been shown for Lip2p (Pignede *et al*. 2000), Lip7p and Lip8p (Fickers *et al*. 2005). *Yarrowia lipolytica* strain with triple deletion of *LIP2*, *LIP7* and *LIP8*, did not have any remaining extracellular lipase activity. Pignede *et al*. (2000) isolated and characterized *LIP2* gene (YALI0A20350g), which encodes an extracellular lipase. Fickers *et al*. (2005) characterized two cell-bound lipases encoded by *LIP7* (YALI0D19184g) and *LIP8* genes (YALI0B09361g). Darvishi (2012) expressed native *LIP2* gene from *Y. lipolytica* DSM3286 and the mutant *LIP2* gene from the mutant *Y. lipolytica* U6 in *S. cerevisiae* and obtained 10 and 15 U/mL lipase activity, respectively. In this study, we expressed these three lipases in different combinations in *S. cerevisiae* and evaluated their activity and effect on lipid and on β-carotene accumulation.

## MATERIALS AND METHODS

### Strains and media

*Escherichia coli* DH5α (Gibco-BRL, Rockville, MD) was used for cloning and vector propagation. *Escherichia coli* cells were grown at 37°C with agitation at 300 rpm in Lysogeny Broth (LB) liquid medium (10 g/L tryptone, 5 g/L yeast extract and 10 g/L NaCl). Transformants were selected on LB medium containing 20 g/L agar and 100 mg/L ampicillin at 37°C.

*Saccharomyces cerevisiae* CEN.PK113–7D strain was a gift from Professor Peter Kötter (Johann Wolfgang Goethe University Frankfurt, Germany). Yeast cells were grown in yeast peptone dextrose (YPD) medium, which contained per liter: 10 g yeast extract, 20 g peptone and 20 g dextrose. YPD was supplemented for selection with 200 mg/L G418 sulfate and 100 mg/L nourseothricin (Stovicek *et al*. 2015).

Yeast nitrogen base (YNB) and mineral salts medium (MSM) media were used for lipases and lipid production. YNB medium contained 10 g dextrose and 6.7 g yeast nitrogen base without amino acids (Sigma–Aldrich, Darmstadt, Germany). The mineral medium (MM1) contained per liter 1 g of NH_4_Cl, 5 g KH_2_PO_4_, 0.1 g MgSO_4 _× 7H_2_O, 5 mg of Fe(SO_4_)_2_, 1 mL of trace elements solution contained 23 mg MnCl_2 _× 2H_2_O, 30 mg MnCl_4 _× H_2_O, 31 mg H_3_BO_3_, 36 mg CoCl_2_.6H_2_O, 10 mg CuCl_2 _× 2H_2_O, 20 mg NiCl_2 _× 6H_2_O, 50 mg ZnCl_2_, 30 mg Na_2_MoO_4 _× 2H_2_O per liter (Merck, Darmstadt, Germany). A total of 10 mL olive oil or triolein as a carbon source was added to the MSM medium. A total of 20 g agar was added to each media for making solid media (Sigma–Aldrich; Hasanuzzaman *et al*. 2004). For β-carotene production, the strains were cultivated in mineral medium (MM2), YNB and YPD media containing olive oil. The mineral medium 2 (MM2) composition was previously described in Jensen *et al*. (2014).

### Vectors

The EasyClone-MarkerFree backbone vectors, pCfB3035 (X-4 MarkerFree), pCfB2904 (XI-3 MarkerFree) and pCfB2909 (XII-5 MarkerFree) were used for cloning the *Y. lipolytica LIP* genes (Table 1). The gRNA vectors, pCfB3047, pCfB3045, pCfB3050 and pCfB3052 were used for integrating the vectors containing *LIP2*, *LIP7* and *LIP8* expression cassettes into *S. cerevisiae* genome (Table 1; Stovicek *et al*. 2015; Jessop-Fabre *et al*. 2016).

**Figure 3.**
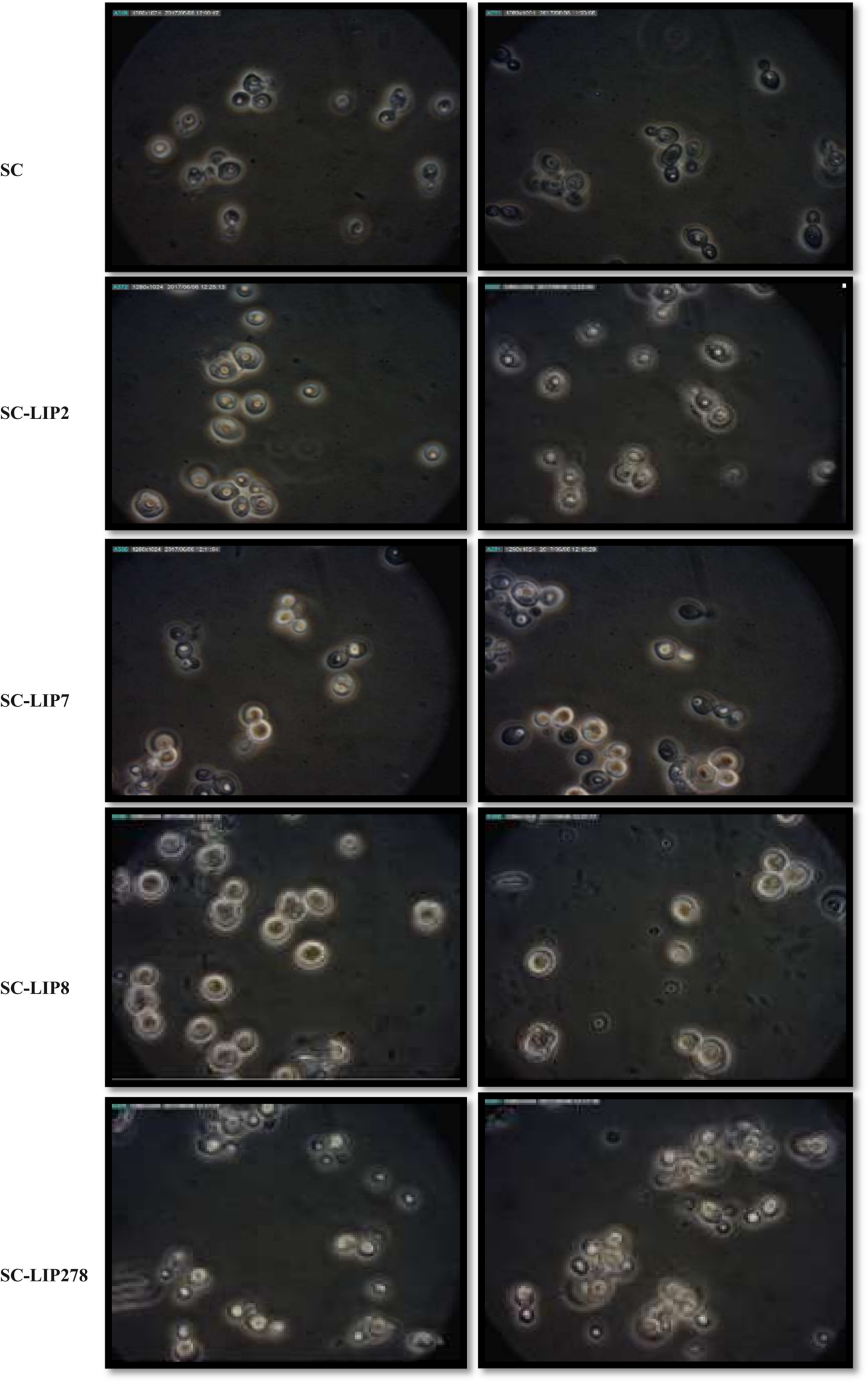
Phase-contrast microscopic images of the parental (SC) and engineered strains (SC-LIP2, SC-LIP7, SC-LIP8 and SC-LIP278) of *S. cerevisiae* in hydrophobic substrates after 4 days. Panels A: Olive oil medium (1% olive oil in mineral salts medium); Panels B: Triolein medium (1% triolein in mineral salts medium). The lipid bodies inside the yeast cells were stained by Oil Red O.

**Table 1.** List of vectors.

Name	Description	Reference
EasyClone-MarkerFree vectors		
pCfB2903	Backbone vector for integration into XI-2 locus of *S. cerevisiae*	Jessop-Fabre *et al*. (2016)
pCfB2904	pXI-3-URA3-DR	Jessop-Fabre *et al*. (2016)
pCfB2909	pXII-5-DR-KlURA3	Jessop-Fabre *et al*. (2016)
pCfB3034	Backbone vector for integration into X-3 locus of *S. cerevisiae*	Jessop-Fabre *et al*. (2016)
pCfB3035	Mutated PAM site in pCfB2901	Jessop-Fabre *et al*. (2016)
pCfB3039	Backbone vector for integration into XII-2 locus of *S. cerevisiae*	Jessop-Fabre *et al*. (2016)
Cas9 expression vector		
pCfB2312	TEF1p-Cas9-CYC1t_kanMX, Episomal plasmid for Cas9 expression	Stovicek *et al*. (2015)
Single target gRNA helper vectors		
pCfB3042	gRNA sequence for targeting site X-4 USER cloned into pCfB2926	Jessop-Fabre *et al*. (2016)
pCfB3045	gRNA sequence for targeting site XI-3 USER cloned into pCfB2926	Jessop-Fabre *et al*. (2016)
pCfB3050	gRNA sequence for targeting site XII-5 USER cloned into pCfB2926	Jessop-Fabre *et al*. (2016)
pCfB3051	gRNA sequence for targeting sites X-3, XI-2 and XII-2 USER cloned into pCfB2926	Jessop-Fabre *et al*. (2016)
pCfB3052	gRNA sequence for targeting sites X-4, XI-3 and XII-5 USER cloned into pCfB2926	Jessop-Fabre *et al*. (2016)
Constructed vectors containing lipase genes		
pCfB4531	Constructed by insertion of *LIP2* gene into pCfB3035	This study
pCfB4533	Constructed by insertion of *LIP7* gene into pCfB2904	This study
pCfB4535	Constructed by insertion of *LIP8* gene into pCfB2909	This study
Constructed vectors containing carotenogenic genes		
pCfB9667	Constructed for expression of *crtYB* using pCfB3034 and BioBricks BB0009 and BB01567.	This study
pCfB9668	Constructed for expression of *crtI* using pCfB2903 and BioBricks BB0410 and BB1568.	This study
pCfB9669	Constructed by for expression of *crtE* using pCfB3039 and BioBricks BB0009 and BB01569.	This study

### Vector construction

Genomic DNA from *Y. lipolytica* strain DSM3286 (DSMZ-German collection) was extracted using the ZR Fungal/Bacterial DNA MiniPrep extraction kit (Zymo Research, Irvine, California, USA) and used as the template for PCR amplification. The primers were designed on the basis of *Y. lipolytica* lipase genes in the NCBI database (Table 2). PCR products were purified from agarose gel and cloned into expression vectors using USER cloning (Jessop-Fabre *et al*. 2016). Glyceraldehyde-3-phosphate dehydrogenase (*TDH3*) promoter of *S. cerevisiae* was used for expressing these genes (McAlister and Holland 1985). The cloning was verified by Sanger sequencing. The purified *LIP2*, *LIP7* and *LIP8* genes and also the cloning vectors included PCFB3035, PCFB2904 and PCFB2909 were digested with *AsiS*I and *Nb.Bsm*I.

**Table 2.** List of primers for amplification of genes and verification of vectors and strains.

Name	Sequence (5′ ˍ 3′)*	Description
*LIP2*_fw	5′-AGTGCAGGUAAAACA**ATG**AAGCTTTCCACCATCCTCTTCAC-3′	Fwd primer for amplification of *LIP2* gene of *Y. lipolytica*
*LIP2*_rv	5′-CGTGCGAU**TTA**GATACCACAGACACCCTCGGTG-3′	Rev primer for amplification of *LIP2* gene of *Y. lipolytica*
*LIP7*_fw	5′-AGTGCAGGUAAAACA**ATG**GTCAGCTTTGGAGCTCGAATC-3′	Fwd primer for amplification of *LIP7* gene of *Y. lipolytica*
*LIP7*_rv	5′-CGTGCGAU**TTA**GTTGGAGAGCTCGAGACCCTC-3′	Rev primer for amplification of *LIP7* gene of *Y. lipolytica*
*LIP8*_fw	5′-AGTGCAGGUAAAACA**ATG**GTATCCCTCTCTGCTCGAATC-3′	Fwd primer for amplification of *LIP8* gene of *Y. lipolytica*
*LIP8_rv*	5′-CGTGCGAU**TCA**GTTCTCAACTTGTGGGGTG-3′	Rev primer for amplification of *LIP8* gene of *Y. lipolytica*
*PR-7039*	5′-ATCTGTCAUATGACGGCTCTCGCATATTA-3′	Fwd primer for amplification of BB01567 (XdCrtYB→)
*PR-7040*	5′-CACGCGAUTTACTGCCCTTCCCATCCGC-3′	Rev primer for amplification of BB01567 (XdCrtYB→)
*PR-7041*	5′-AGTGCAGGUATGGGAAAAGAACAAGATCAGG-3′	Fwd primer for amplification of BB01568 (XdCrtI←)
*PR-7042*	5′-CGTGCGAUTCAGAAAGCAAGAACACCAACG-3′	Rev primer for amplification of BB01568 (XdCrtI←)
*PR-7043*	5′-ATCTGTCAUATGGATTACGCGAACATCCTC-3′	Fwd primer for amplification of BB01569 (XdCrtE→)
*PR-7044*	5′-CACGCGAUTCACAGAGGGATATCGGCTAG-3′	Rev primer for amplification of BB01569 (XdCrtE→)
PR-22 406	5′-CGTGCGAUGGAAGTACCTTCAAAGAATGG-3′	Fwd primer for amplification of BB0009 (PPGK1→)
PR-22 407	5′-ATGACAGAUTTGTTTTATATTTGTTGTAAAAAGTAG-3′	Rev primer for amplification of BB0009 (PPGK1→)
PR-1852	5′-CACGCGAUATAAAAAACACGCTTTTTCAG-3′	Fwd primer for amplification of BB0410 (PTDH3←)
PR-1853	5′-ACCTGCACUTTTGTTTGTTTATGTGTGTTTATTC-3′	Rev primer for amplification of BB0410 (PTDH3←)
ID2221	5′-GTTGACACTTCTAAATAAGCGAATTTC-3′	Universal primer anneals to any EasyClone vectors
ID2220	5′-CCTGCAGGACTAGTGCTGAG-3′	Universal primer anneals to any EasyClone vectors
ID905	5′-CTCACAAAGGGACGAATCCT-3′	Verifies UP region of chromosome site X-4 with ID2221
ID911	5′-GTGCTTGATTTGCGTCATTC-3′	Verifies UP region of chromosome site XI-3 with ID2221
ID900	5′-GTGGGAGTAAGGGATCCTGT-3′	Verifies Down region of chromosome site XII-5 with ID2220

*Start and stop codons are marked in bold, translational enhancer (Kozak) sequence is underlined.

**Table 3.** List of BioBricks used for construction of β-carotene plasmids.

ID	Description
**BB01567**	Gene *crtYB* from *Xanthophyllomyces dendrorhous*
**BB01568**	Gene *crtI* from *Xanthophyllomyces dendrorhous*
**BB01569**	Gene *crtE* from *Xanthophyllomyces dendrorhous*
**BB0009**	Promoter PPGK1 from *S. cerevisiae*
**BB0410**	Promoter PTDH3 from *S. cerevisiae*

The recombinant plasmids were transformed into *E. coli* DH5a, and cultured on LB plates containing 25 µg/mL ampicillin as a selection marker. The positive transformants were grown in liquid LB medium containing 25 µg/mL ampicillin overnight and used for extraction of recombinant plasmids. The integrative vectors were linearized by FastDigest *Not*I (Life Technologies, Waltham, Massachusetts, USA) restriction enzyme and purified from agarose gel before transformation into *S. cerevisiae*.

For the construction of β-carotene vectors, the carotenogenic genes encoding phytoene synthase/lycopene cyclase (*crtYB*), phytoene desaturase (*crtI*) and geranylgeranyl diphosphate synthase (*crtE*) from the red yeast *X. dendrorhous* were obtained from AddGene (Verwaal *et al*. 2007). The vectors, primers and BioBricks used are listed in Tables 1, 2 and 3, respectively. The β-carotene BioBricks were amplified by PCR using Phusion U polymerase (Thermo Fisher Scientific, Waltham, MA) under the following conditions: 98°C for 30 s; 6 cycles of 98°C for 10 s, 51°C for 20 s and 72°C for 30 s/kb; 26 cycles of 98°C for 10 s, 58°C for 20 s and 72°C for 30 s/kb and 72°C for 5 min. The BioBricks were purified from agarose gel using the NucleoSpin®Gel and PCR Clean-up kit (Macherey-Nagel, Düren, Germany). The parental vectors were digested with FastDigest AsiSI (Thermo Fisher Scientific) and nicked with Nb.BsmI (New England Biolabs, Hitchin, United Kingdom). Next, the BioBricks were incubated in CutSmart® buffer (New England Biolabs) with USER enzyme and the parental vector for 25 min at 37°C, followed by 10 min at 25°C, 10 min at 20°C and 10 min at 15°C. The USER reactions were transformed into competent *E. coli* DH5a and selected, as described above.

### Strain construction

The *S. cerevisiae* cells were transformed by PEG/LiAc method (Gietz and Schiestl 2007). First, *cas9* endonuclease expression plasmid (pCfB2312) was transformed into competent *S. cerevisiae* cells and transformants were selected on YPD plates with 200 mg/L G418 sulfate. Then the resulting strain expressing *cas9* was used for further transformations with integration elements and helper gRNA vectors (Jessop-Fabre *et al*. 2016). The transformants were selected on YPD plates supplemented with 200 mg/L G418 sulfate and 100 mg/L nourseothricin. The correct integration was confirmed by colony-PCR using integration site-specific primers (Tables 2 and 4).

### Assay of lipase production

Qualitative assay of lipase production was performed on MSM plates containing tributyrin (10 mL/L) by determination of the hydrolysis halo (H) diameter to the cell colony (C) diameter (Pignede *et al*. 2000).

Quantitative assay of lipase production was done using *p*-nitrophenyl laurate, *p*-palmitate, *p*-decanoate and *p*-hexanoate substrates by spectrophotometric method (Fickers *et al*. 2003; Fickers *et al*. 2011). The *p*-nitrophenyl laurate and *p*-palmitate are specific substrates for Lip 2. The *p*-decanoate and *p*-hexanoate are recognized as specific substrates for Lip 8 and Lip 7, respectively (Fickers *et al*. 2003, 2005, 2011).

### Determination of yeast cell growth

The expression of heterologous extracellular and cell-bound lipases are essential for the growth of *S. cerevisiae* in oily media. The yeast strains (control and engineered strains) were pre-cultured in the YPD medium. The overnight culture was inoculated in 50 mL YNB and MSM media containing 1% olive oil or triolein and incubated at 30°C and 150 rpm. The yeast biomass was harvested by at 11 000 rpm for 5 min and washed twice with ice-cold acetone to remove oily substrates and dried in the oven at 60°C for 24 h. The samples were used for DCW measurements and intracellular lipid extraction (Darvishi, Salmani and Hosseini 2019).

### Analysis of yeast lipid bodies

The yeast cells were stained using Oil Red O to the visualization of lipid bodies. The cells were harvested by centrifugation at 11 000 rpm for 5 min and washed twice, next, they were resuspended in phosphate-buffered saline (PBS). The cell pellet was fixed by 4% formaldehyde in PBS for 20 min and washed twice with PBS. The cells were stained with 0.2% Oil Red O in a water-isopropanol (1:1 v/v) solution for 15 min at room temperature in the dark place. After that, they were washed two times with PBS for removing the surplus dye and resuspended in 50 mM sodium phosphate buffer (pH 6.8; Rossana *et al*. 2011; Shockey *et al*. 2011). Samples were observed by an Olympus BX 41 phase-contrast microscope. The images were recorded by Dino-Eye USB Dia-22 mm and acquisition software DinoCapture 2.0 (DinoCapture, Tokyo, Japan).

### Intracellular lipid extraction

The extraction of intracellular lipids was performed according to a previously described method with some modifications (Darvishi, Salmani and Hosseini 2019). A total of 1 gram of dried cells was added into 10 mL of 3N HCl (Sigma–Aldrich, Darmstadt, Germany) and heated to boiling and immediately cooled on ice. Then 30 mL chloroform-methanol (2:3 v/v) was added to the sample. After incubation at 37°C for 1 h, the chloroform–methanol phase was collected and dried at 80°C by rotary evaporator. The lipid content (%) was calculated as intracellular lipid weight (mg) per DCW (g) (Shi *et al*. 2014). The composition of fatty acid was determined by gas chromatography-mass spectrometry (GC–MS). (Shi *et al*. 2014; Valle-Rodríguez *et al*. 2014).

### Carotenoid extraction

For β-carotene extraction, 0.5 mL of the cultivation broth was transferred into a 2 mL microtube (Sarstedt, Numbrecht, Germany). Each sample was centrifuged at 10 000x *g* for 10 min, and the supernatant was removed. Next, 0.5 mL of 0.5–0.75 mm acid-washed glass beads were added to each tube, followed by the addition of 0.5 mL of ethyl acetate supplemented with 0.01% 3,5-di-tert-4- butylhydroxytoluene (BHT). The BHT was added to prevent carotenoid oxidation. The cells were disrupted using a Precellys R 24 homogenizer (Bertin Corp., Montigny-le-Bretonneux, France) in four cycles of 5500 rpm for 20 s. The tubes were placed on ice for 1 min in between each lysis cycle to cool down and avoid product degradation. After disruption, the cells were centrifuged for 10 min at 10 000 x *g*. For the quantification of β-carotene by HPLC, 100 μL of the solvent fraction was transferred to HPLC vials. For DCW measurements, 1 mL of the cultivation broth was transferred into a pre-weighed 2 mL microtube (Sarstedt, Numbrecht, Germany). The tubes were centrifuged at 10 000 x *g* for 10 min. The supernatant was removed and the samples were washed with 1 mL of sterile water. The tubes with biomass pellets were dried at 60°C for 96 h and weighed on an analytical scale.

### Carotenoid quantification by HPLC

For HPLC analysis, the procedure was as described by Kildegaard *et al*. (2017). A total of 100 μL of ethyl acetate extract was evaporated on SpeedVac, and the dry extracts were redissolved in 1 mL 99% ethanol + 0.01% BHT. Then, the extracts were analyzed by HPLC (Thermo Fisher Scientific) equipped with a Discovery HS F5 150 mm x 2.1 mm column (particle size 3 mm). The column oven temperature was set to 30°C. All organic solvents used were HPLC grade (Sigma Aldrich, St. Louis, MO). The flow rate was set to 0.7 mL/min with an initial solvent composition of 10 mM ammonium formate (pH = 3, adjusted with formic acid; solvent A) and acetonitrile (solvent B; 3:1) until minute 2.0. Solvent composition was then changed at minute 4.0 following a linear gradient until % A = 10.0 and % B = 90.0. The solvent composition was kept until 10.5 min when the solvent was returned to initial conditions, and the column was re-equilibrated until 13.5 min. The injection volume was 10 µL. The peaks obtained from the sample analysis were identified by comparison to prepared standards and integration of the peak areas was used to quantify carotenoids from obtained standard curves. β-carotene was detected at a retention time of 7.6 min by measuring absorbance at 450 nm. The β-carotene standard (C4582–5 mg) was purchased from Sigma-Aldrich.

## RESULTS

### Lipase activity in the engineered yeast strains

The *LIP2*, *LIP7* and *LIP8* from *Y. lipolytica* were expressed either individually or in combination in *S. cerevisiae* CEN.PK113–7D (SC). The engineered yeast strains expressing individual lipases were named SC-LIP2, SC-LIP7 and SC-LIP8. The strain SC-LIP278 expressed all three lipase genes. All engineered yeasts produced heterologous lipases and halo around yeast colonies were observed on YNB tributyrin agar after 3-day incubation. Strains SC-LIP2 and SC-LIP278 produced larger halos than SC-LIP7 and SC-LIP8 strains, whereas no halo was formed around the colony of control strain SC since it does not have any extracellular lipase activity (Figure S1, Supporting Information). Larger halo around SC-LIP2 and SC-LIP278 is consistent with the high extracellular lipase activity of Lip2 (Fickers *et al*. 2005).

**Figure 1.**
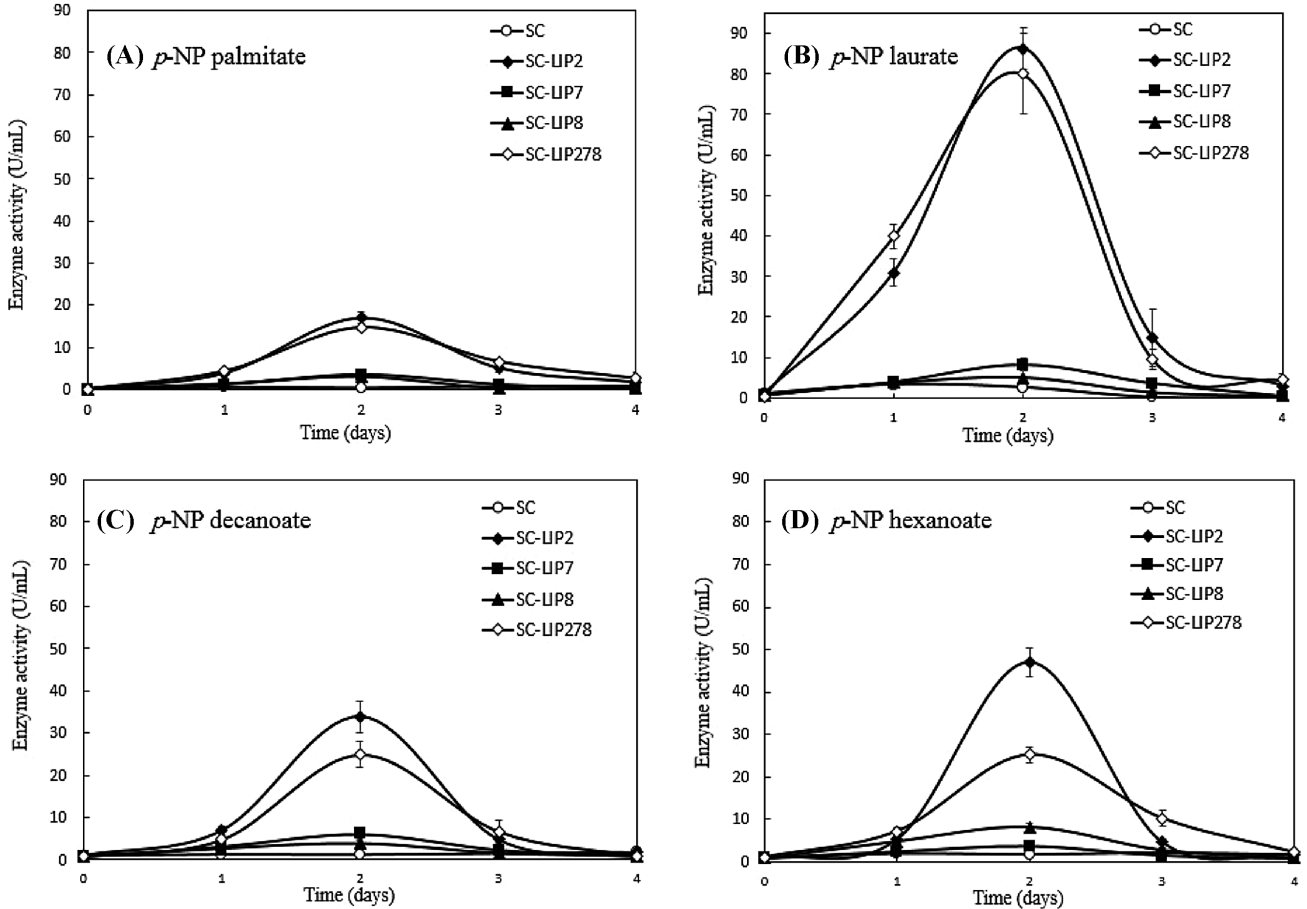
Lipase activity of the parental (SC) and engineered (SC-LIP2, SC-LIP7, SC-LIP8 and SC-LIP278) strains of *S. cerevisiae* during 4 days in the YNB medium. Lipase activity was evaluated using *p*-nitrophenyl esters with different carbon chain lengths as lipase substrates: **(A)** C16 (*p*-NP palmitate), **(B)** C12 (*p*-NP laurate), **(C)** C10 (*p*-NP decanoate) and **(D)** C6 (*p*-NP hexanoate). Error bars in the graph represent the mean standard error from three independent experiments.

The lipase activity was then measured on several defined substrates, *p*-nitrophenyl esters with carbon chain lengths of 6, 10, 12 and 16 carbons (Fig. 1). *p*-Nitrophenyl palmitate (C_16_) and *p*-nitrophenyl laurate (C_12_) were preferred substrates for Lip2, reaching 85 U/mL for SC-LIP2 strain on C12 substrate. *p-*Nitrophenyl decanoate (C_10_) and *p*-nitrophenyl hexanoate (C_6_) were preferred substrates for Lip7 and Lip8, respectively.

**Figure 2.**
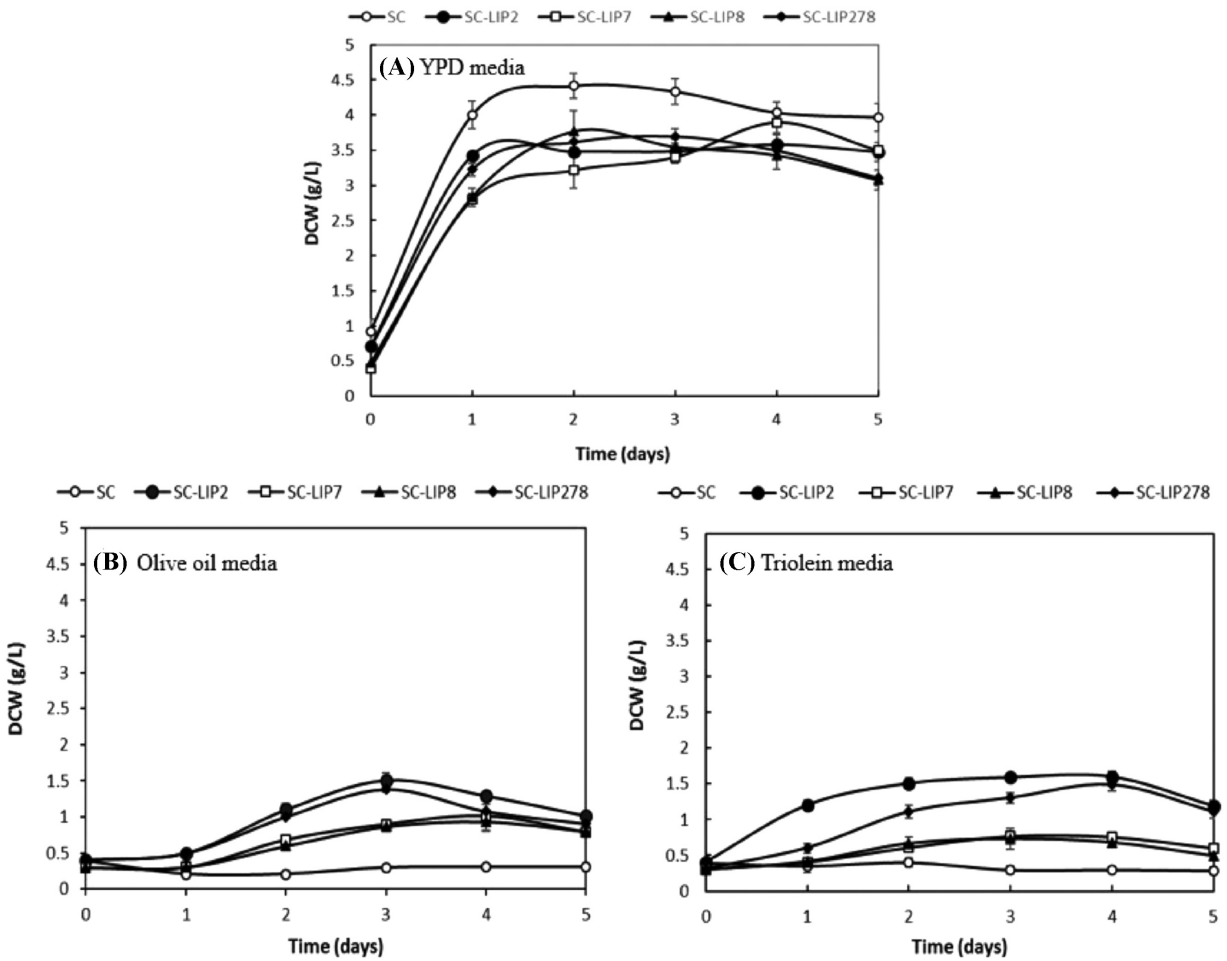
The growth of the parental (SC) and engineered (SC-LIP2, SC-LIP7, SC-LIP8 and SC-LIP278) strains of *S. cerevisiae* in **(A)** YNB medium (1% dextrose in YNB medium), **(B)** Olive oil medium (1% olive oil in mineral salts medium) and **(C)** Triolein medium (1% triolein in mineral salts medium). Error bars in the graph represent the mean standard error from three independent experiments.

The growth of the engineered strains was evaluated in liquid medium with 1% olive oil and triolein as the sole carbon source (Fig. 2 and Figure S2, Supporting Information). Under these conditions, all the engineered strains grew to at least 0.6 g DCW/L. The highest growth was observed for SC-LIP2 and SC-LIP278 strains that reached 1.4–1.5 g DCW/L after 4 days (Fig. 2). Wild-type strains of *S. cerevisiae* (SC) cannot produce any extracellular lipase and they are unable to use trioleine or olive oil as carbon source. However, some TAGs may hydrolyze during the autoclaving of the medium, and the resulting free fatty acids and glycerol can support the very slow growth by SC strain. The final DCW of the SC was 0.33 g DCW/L in trioleine medium and 0.35 g DCW/L in the olive oil medium (Fig. 2), which was sufficient for measuring the lipid content.

### Lipid accumulation in the engineered yeast strains

A significant difference in lipid accumulation was observed between SC and the engineered strains. When the strains were grown on triolein medium, the lipid content was 4–6-fold higher in lipase-expressing strains than in control strain. The highest lipid content was in strain SC-LIP278, 38.2% of DCW in olive oil and 41.1% of DCW triolein (Table 5). Phase-contrast microscopic images confirmed the increased lipid accumulation (Fig. 3 and Figure S3, Supporting Information). The engineered *S. cerevisiae* strains were able to consume oily substrates and accumulate fatty acids in lipid bodies (Fig. 3). The major accumulated fatty acids in the yeast strains were myristic (C14:0), palmitic (C16:1), oleic (C18:1) and linoleic (C18:2) acids (Table 5).

**Table 4.** List of yeast strains.

Strain name	Parental strain	Description	Reference
*Y. lipolytica* DSM3286	-	Native strain	Darvishi (2012)
*S. cerevisiae* CEN.PK113–7D	-	*MATa URA3 HIS3 LEU2 TRP1 MAL2–8c SUC2*	Jessop-Fabre *et al*. (2016)
ST7574	CEN.PK113–7D	Strain CEN.PK113–7D transformed with pCfB2312	Milne *et al*. (2019)
*S. cerevisiae* SC-LIP2	*S. cerevisiae* CEN.PK113–7D	X-4:TDH3p-*LIP2-*ADH1t	This study
*S. cerevisiae* SC-LIP7	*S. cerevisiae* CEN.PK113–7D	XI-3:TDH3p-*LIP7-*ADH1t	This study
*S. cerevisiae* SC-LIP*8*	*S. cerevisiae* CEN.PK113–7D	XII-5:TDH3p-*LIP8-*ADH1t	This study
*S. cerevisiae* SC-LIP278	*S. cerevisiae* CEN.PK113–7D	X-4:TDH3p-*LIP2-*ADH1t/XI-3:TDH3p-*LIP7-*ADH1t/XII-5:TDH3p-*LIP8-*ADH1t	This study
ST8936	ST7574	*XdCrtl XdCrtYB XdCrtE*	Milne *et al*. (2019)
ST9754	*S. cerevisiae* SC-LIP278	Strain SC-LIP278 transformed with pCfB9667, pCfB9668 and pCfB9669.	This study

**Table 5.** Analysis of the fatty acid composition of the yeast strains grown on various carbon sources after 4 days.

Medium^a^	Strain	C12:0	C14:0	C16:1	C18:1	C18:2	Total
Olive oil	**SC**	-	2.6 ± 0.3	2.4 ± 0.3	1.3 ± 0.2	0.2 ± 0.1	6.5 ± 0.9
	**SC-LIP2**	0.3 ± 0.1	6.3 ± 0.4	6 ± 0.3	10 ± 1.1	2 ± 0.2	24.6 ± 2.1
	**SC-LIP7**	0.2 ± 0.1	10 ± 1.1	8 ± 0.7	10 ± 1.2	n.d.	28.2 ± 3.1
	**SC-LIP8**	0.5 ± 0.2	14.5 ± 1.6	10.3 ± 1.2	10 ± 1.1	n.d.	35.3 ± 4.1
	**SC-LIP278**	1.2 ± 0.3	9 ± 0.9	10.4 ± 0.8	16 ± 1.6	1.6 ± 0.4	38.2 ± 4
Triolein	**SC **	0.9 ± 0.2	1.1 ± 0.2	2 ± 0.3	2.3 ± 0.4	n.d.	6.3 ± 1.1
	**SC-LIP2**	0.3 ± 0.1	3.5 ± 0.3	5.6 ± 0.3	15.2 ± 1.7	4 ± 0.3	28.6 ± 2.7
	**SC-LIP7**	0.3 ± 0.1	5.7 ± 0.4	6.1 ± 1	15.5 ± 1.7	n.d.	27.6 ± 3.2
	**SC-LIP8**	0.7 ± 0.2	5.4 ± 0.6	7.5 ± 0.9	21.5 ± 2.3	1.3 ± 0.3	36.4 ± 4.3
	**SC-LIP278**	0.8 ± 0.2	3.1 ± 0.2	5.7 ± 0.5	28.3 ± 2.5	3.2 ± 0.3	41.1 ± 3.7

The fatty acid composition is expressed as each fatty acid (w)/dry cell weight (w) × 100.

C12:0, Lauric acid; C14:0, Myristic acid; C16:1, Palmitoleic acid; C18:1, Oleic acid and C18:2, Linoleic acid.

a Strains were grown on MSM with 1% olive oil and MSM with 1% oleic acid.

### Expression of heterologous genes for the production of β-carotene by engineered strain SC-LIP278

**Figure 4.**
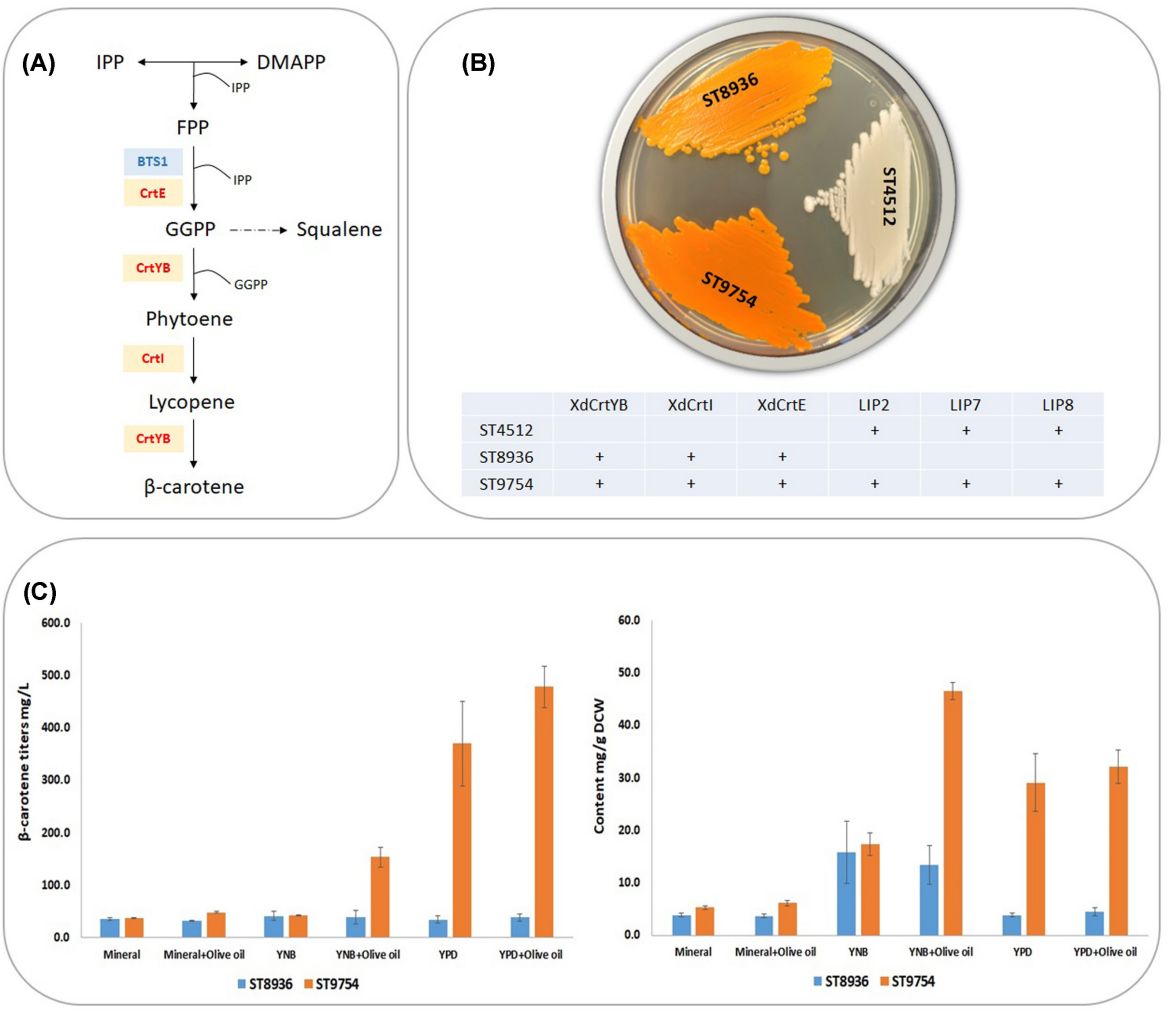
β-carotene production by engineered strains. **(A)** Heterologous β-carotene pathway introduced in *S. cerevisiae*. Blue boxes indicate native genes; yellow boxes indicate inserted genes. IPP: Isopentenyl pyrophosphate; DMAPP: Dimethylallyl pyrophosphate; FPP: Farnesyl pyrophosphate; GGPP; geranylgeranyl pyrophosphate; BTS1 and CrtE: geranylgeranyl pyrophosphate synthase; CrtI: phytoene desaturase; CrtYB: phytoene synthase and lycopene cyclase. **(B)** Phenotype and Genotype of strains SC-LIP278 (ST4512), ST8936 and ST9754. **(C)** β-carotene titers and yield of strains ST8936 and ST9754 cultivated for 72 h in Mineral, YNB and YPD media supplemented with 1% v/v olive oil. The error bars represent standard deviations calculated from biological triplicate experiments.

To produce β-carotene in *S. cerevisiae*, the expression of heterologous carotenogenic genes is necessary. *S. cerevisiae* naturally produces the precursor geranylgeranyl pyrophosphate (GGPP) via GGPP synthase Bts1p. Several studies have shown that GGPP synthases from natural producers, such as CrtE from *X. dendrorhous*, have higher activity than *S. cerevisiae* Bts1p (Chemler, Yan and Koffas 2006; Verwaal *et al*. 2007; Xie *et al*. 2015). Therefore, we chose to express three genes from *X. dendrorhous* in *S. cerevisiae* strain SC-LIP278: *crtE* (GGPP synthase), *crtYB* (phytoene synthase and lycopene cyclase) and *crtI* (phytoene desaturase; Fig. 4A).

The resulting strain SC-LIP278*β* (ST9754) was cultivated for 72 h in mineral, YNB and YPD media with or without 1% v/v olive oil. Strain SC-LIP278 was the non-producing control, while β-carotene strain ST8936 from Milne, Tramontin and Borodina (2019) was the control strain without lipases. When grown on YPD plate, the strain expressing lipases had a more intense orange color than the strain with carotenogenic genes only (Fig. 4B). The strain with lipases produced 4- to 12-fold higher titer of β-carotene than the strain without lipases on YNB with olive oil, YPD and YPD with olive oil (Fig. 4C). The highest β-carotene content was 46.5 mg/g DCW in YNB with olive oil. Surprisingly, the lipase-containing strain also produced ca. 10-fold more β-carotene than control strain on YPD medium without oil. This may be explained by the presence of the small amount of fats in meat peptone used for YPD medium preparation (Taskin and Kurbanoglu 2011).

## DISCUSSION

The increasing customer demand for natural β-carotene has encoraged the development of microbial processes for β-carotene production. One of the potential microbial cell factories is the baker's yeast *S. cerevisiae*. The yeast is already widely used in industrial biotechnology for the production of fuels and chemicals (Li and Borodina 2015). In this study, we hypothesized that increasing lipid accumulation in *S. cerevisiae*, like in *Y. lipolytica* (Gao *et al*. 2017; Larroude *et al*. 2017), could be an effective strategy to improve the production of β-carotene. For this, we integrated lipase-coding genes *LIP2*, *LIP7*, *LIP8* from *Y. lipolytica* into the *S. cerevisiae* genome individually or in combination. This highest lipase activity of 85 U/mL was measured in the broth of *LIP2*-expressing strain after 2 days of cultivation. This activity is 5-fold higher than measured in another study for the wild-type *Y. lipolytica* (Darvishi 2014). The high activity can be explained by the use of a strong constitutive *TDH3* promoter for driving *LIP2* expression in the engineered *S. cerevisiae* (McAlister and Holland 1985). Shockey *et al*. (2011) have expressed the codon-optimized *LIP2* under control of the promoters galactose-inducible *GAL1* and fatty acid-inducible *PEX11* with some modifications in *S. cerevisiae*. This study showed that codon optimization does not positively contribute to the enzyme production or activity, the lipase activity was around 1.3 U/mg. In another work, Darvishi (2012) expressed the native *LIP2* gene from *Y. lipolytica* DSM3286 and the mutant *LIP2* gene from the mutant *Y. lipolytica* U6 in *S. cerevisiae* strain CEN.PK 113–5D under *GPD* promoter and their lipase activity were 10 and 15 U/mL, respectively.

The engineered *S. cerevisiae* strains were able to efficiently use and grow on olive oil and triolein as hydrophobic carbon sources (Fig. 2). Also, lipid accumulation and the lipid content was 4–6-fold higher in lipase-expressing strains than in control strain when the strains were grown on triolein medium (Table 5). Valle-Rodríguez *et al*. (2014) enhanced fatty acids accumulation by 4-fold in *S. cerevisiae* via gene deletions related to the syntheses of TAGs and utilization of fatty acids pathways such as *DGA1*, *LRO1*, *ARE1* and *ARE2*. Additionally, fatty acid production was enhanced to 5-fold by combining the disruption of β-oxidation and syntheses of TAGs and SEs pathways. The yeast fatty acid content and composition are important if the whole yeast biomass would be used for feed or food (Shi *et al*. 2014). The major fatty acids accumulated in the engineered strains are myristic, palmitic, oleic and linoleic acid. The composition of fatty acids in SC-LIP278 after 4 days growing in triolein was as follows: C12:0 (0.8%), C14:0 (3.1%), C16:1 (5.7%), C18:1 (28.3%) and C18:2 (3.2%) (Table  5). The profile was similar to the previous study by Valle-Rodríguez *et al*. (2014). They found a slightly higher proportion of C_18_ fatty acids (31.5% w/w) and a lower proportion of C_14_ and C_16_ fatty acids in the storage neutral lipids (Table 5). The accumulation of C_18_ fatty acids in *Y. lipolytica* is around 22% (w/w), and this difference in lipids composition could be caused by the alteration of host cell physiology and to the different types of internal lipases in the strains.

After engineering *S. cerevisiae* for expression of *LIP2*, *LIP7* and*LIP8*, we rewired the strain for the production of β-carotene and tested its performance in different cultivation media containing oil. By using this strategy, we achieved the best β-carotene titer of 477.9 mg/L in YPD medium with 1% v/v olive oil, while the highest content of 46.5 mg/g DCW was obtained in YNB medium with 1% v/v olive oil. The highest titer and content were obtained for strain SC-LIP278*β*.

Most of studies have been searching for sustainable and cheaper substrates for the production of β-carotene. In work done by Cheng *et al*. (2020), xylose derived from bioenergy sorghum was used as the substrate for the production of β-carotene. The authors used a xylose high-concentrated hydrolysate (66 g xylose/L) to obtain a titer of 114.50 mg/L of β-carotene in 5 mL culture tubes. The engineered *S. cerevisiae* strain expressed the heterologous carotenogenic genes *crtE*, *crtI* and *crtYB* from *X. dendrorhous*. In another work, the effect of seawater was investigated (Guo *et al*. 2019). After cultivating the engineered *S. cerevisiae* strain in synthetic seawater combined with a high carbon-to-nitrogen ratio (C:N = 50), the authors reported a β-carotene production of 10.44 mg/g DCW. The strain used in the study was previously reported by the same research group and engineered using adaptive laboratory evolution (ALE) to improve carotenoid production (Reyes, Gomez and Kao 2014). The utilization of oily substrates for the production of carotenoids has not been well studied, particularly in *S. cerevisiae*, which does not naturally utilize extracellular lipids. Several studies have reported the positive correlation between lipid content and carotenoid production most owing to the hydrophobic nature of carotenoids, which leads to its storage into lipid bodies (Ciegler, Arnold and Anderson 1959; Xie *et al*. 2015; Larroude *et al*. 2017; Guo *et al*. 2019). Therefore, improving the lipid metabolism in *S. cerevisiae* might be a promising strategy to obtain higher titers of β-carotene when using an oily substrate as a carbon source. Sun *et al*. (2016) reported an increase of 130% in β-carotene content when the cultivation medium was supplemented with 60 mg/L of palmitoleic acid. The authors reinforced the necessity of a sufficient supply of precursors in the mevalonate pathway. Their results, however, suggest that optimizing the mevalonate pathway alone is not enough to enhance β-carotene production and that strengthening unsaturated fatty acid biosynthesis or its supplementation in the media might be a promising strategy for optimizing β-carotene biosynthesis. In another work, Nanou and Roukas (2016) studied the impact of waste cooking oil (WCO) as a substrate for β-carotene production. In this work, the authors used the natural producer *Blakeslea trispora* to reach a carotenoid production of 2 g/L, and the major accumulated compound was β-carotene (74.2%).

Other studies have reported the production of β-carotene by *S. cerevisiae* employing different metabolic engineering strategies. (Verwaal *et al*. 2007; Xie *et al*. 2015; Godara *et al*. 2019). Most of the studies have reported the optimization in the mevalonate pathway, squalene downregulation and overexpression of carotenogenic genes as strategies for improvements in carotenoid biosynthesis using different microorganisms (Verwaal *et al*. 2007; Yan *et al*. 2011; Shi *et al*. 2014; Kildegaard *et al*. 2017; Tramontin *et al*. 2019). In this study, the strain ST9754 did not have the mevalonate or the β-carotene pathway optimized. Therefore, a higher titer could be obtained if key enzymes, such as HMG1 and GGPP synthase, were overexpressed to improve the carbon flux towards β-carotene. The results and strategy used in this study show the potential of engineering *S. cerevisiae* to efficiently utilize oil as a carbon source by expressing lipases from oleaginous microorganisms, such as *Y. lipolytica*. Additionally, the engineered strain has the potential to be further cultivated in hydrophobic waste substrates for sustainable and low-cost production of carotenoids. Finally, to our knowledge, this is the first study to report the effects of expressing lipases for the production of β-carotene in *S. cerevisiae*, an approach that led to the highest β-carotene content reported so far in an *S. cerevisiae* strain.

## CONCLUSION

An extracellular lipase (LIP2) and two cell-bound lipases (LIP7 and LIP8) and eight from *Y. lipolytica* were successfully expressed in *S. cerevisiae*. The engineered strains grew on triolein or olive oil as the sole carbon source. The strain, expressing all three lipases and the β-carotene pathway, accumulated 46.5 mg β-carotene/g DCW in yeast nitrogen base medium with added 1% olive oil, which was 3.5-fold higher than the strain without lipases. The study demonstrates the potential of a yeast-based process for β-carotene production with the addition of hydrophobic substrates.
